# Improvements for recording retinal function with Microelectrode Arrays

**DOI:** 10.1016/j.mex.2023.102543

**Published:** 2023-12-29

**Authors:** D.L. Rathbun, A. Jalligampala, E. Zrenner, Z. Hosseinzadeh

**Affiliations:** a Department of Ophthalmology, Detroit Institute of Ophthalmology, Henry Ford Health System, Detroit, MI 48202, USA; b Department of Ophthalmology and Visual Sciences, University of Louisville, Louisville, KY 40202, USA; c Institute for Ophthalmic Research, Eberhard Karls University, 72076 Tübingen, Germany; d Werner Reichardt Centre for Integrative Neuroscience (CIN), 72076 Tübingen, Germany; e Department of Ophthalmology and Eye Hospital, University of Leipzig, Leipzig, Germany; f Department of Ophthalmology, Radboud University Medical Center, Nijmegen, The Netherlands

**Keywords:** Retina, Microelectrode arrays, Electrophysiology, Action potential, Retinal Recoding with Microelectrode Arrays

## Abstract

A microelectrode array (MEA) is a configuration of multiple electrodes that enables the concurrent targeting of multiple sites for extracellular recording and stimulation. By utilizing light pulses or electrical stimulations, MEA recordings unveil the complex patterns of electrical activities that arise from the signaling processes within the retinal network. Here, we present a stepwise approach for using microelectrode arrays (MEAs) for recording action potentials from the mouse retina in response to electrical and light stimuli. We provide detailed techniques accompanied by description of a custom optical system, example recordings, troubleshooting guidelines, and data processing methods including spike sorting and code resources for analyzing light and electrical responses. The comprehensive nature of this paper aims to guide researchers in utilizing MEAs effectively for investigating retinal functionality. In particular, it can be easy to have a MEA experiment fail, but hard to identify the source of the failure. This paper is meant to demystify that process. It includes:

• A description of MEA setup, recording, and spike data validation.
• A troubleshooting guide for common failure modes in MEA recordings from mouse retina.
• Spike detection and sorting to precisely extract distinctive action potential.

## Graphical abstract

**Figure:**
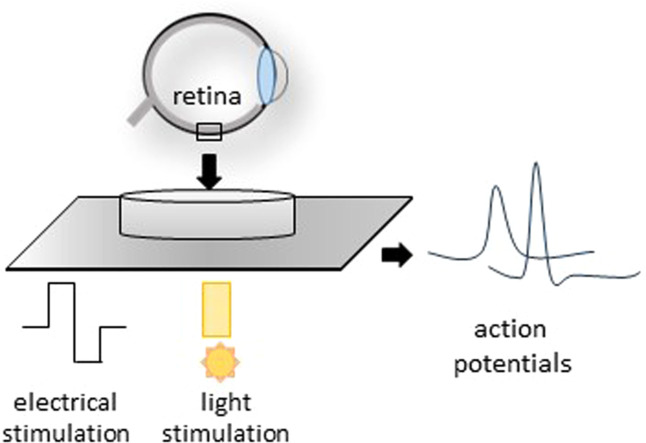

Specifications table

**Table:** 

Subject area:	Neuroscience
More specific subject area:	Electrophysiology
Name of your method:	Retinal Recoding with Microelectrode Arrays
Name and reference of original method:	Meister M, Pine J, Baylor DA [17] Multi-neuronal signals from the retina: acquisition and analysis J Neurosci Methods 51:95–106
Resource availability:	The resources necessary to reproduce this method are presented either in the main paper or the supplement.

## Method details

MEAs serve as neural circuitry interfaces [1,2]. The numerous electrodes of a MEA enable simultaneous measurement of activity from multiple neurons. MEAs complement various other techniques to characterize neuronal activity including: patch clamp measurements [3,4], calcium imaging [[5], [6], [7]], and voltage-sensitive dye imaging [8]. This enables us to understand the dynamics and interactions of neural networks in real-time.

MEA recordings have been extensively validated as a robust and noninvasive technique in retina research [9,10]. Direct and Network-mediated retinal ganglion cell (RGC) responses to light, electrical or chemical stimulation have been recorded using MEAs [[11], [12], [13], [14]]. Recording multiple RGC responses allows for identifying different RGC types in the population and determining their receptive fields [15,16]. While MEAs offer several advantages, it is important to acknowledge their limitations. One limitation shared with other extracellular recording methods is a potential bias in favor of neurons with larger somas which can be recorded on multiple electrodes and from larger distances [10]. In the specific case of electrical stimulation experiments, electrical stimulation artifacts can obscure direct responses from RGCs (<10 ms); therefore, indirect responses of RGCs are the most reliable [17].

**Fig. 1:**
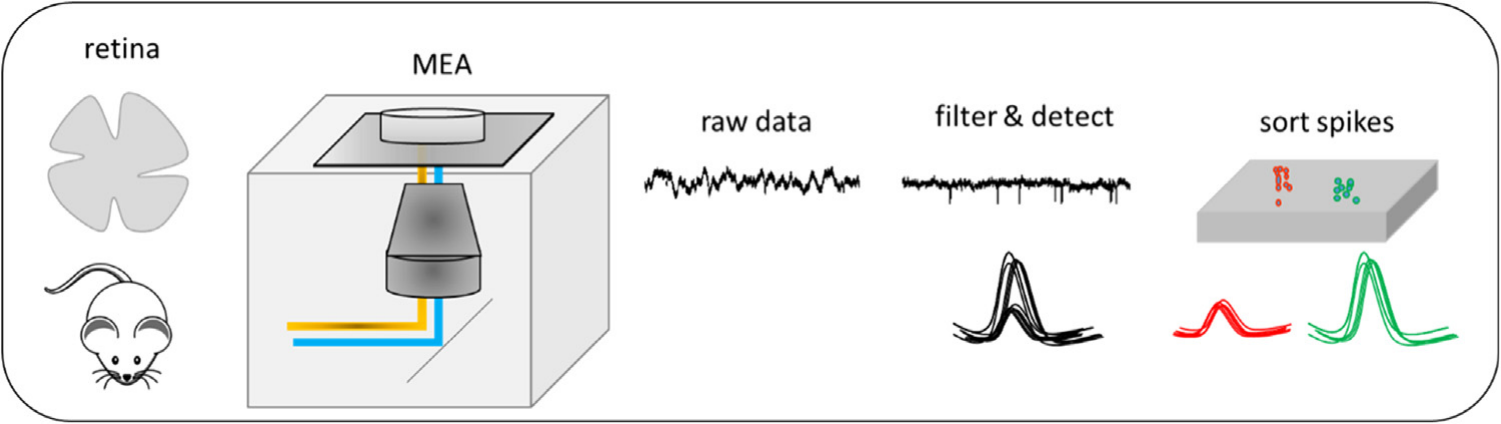
A workflow for recording mouse retina using MEAs, raw data are extracted, filtered and detected as well as spike sorted.

Different types of MEAs have been used for retinal recording. Perforated MEAs, by applying a slight vacuum through the perforations pull the tissue towards the electrode, leading to enhanced contact between the retina and the electrodes which results in an increase in the signal-to-noise ratio and decreases tissue movement during the experiment [18]. High-density MEAs (HDMEAs) can record every ganglion cell in the functional mosaic of a small patch of retina [19] and track the action potential as it travels along individual neurons [20]. MEAs with low impedance make it possible to measure micro electroretinograms (mERGs) that are the visual population responses of different retinal cell classes. Three ERG waves - consisting of small *a*-waves (driven by rod and cone), *b*-waves (driven by ON bipolar) and *c*-waves (driven by photoreceptors and pigment epithelium) - have been reported [12,21]. Despite several groups using MEAs to record from the retina, new investigators still find it difficult to achieve successful recordings using only the published literature as a guide. To address that difficulty, in this article, we describe our MEA recording setup, retina preparation, electrical and visual stimulation, data collection, data processing (spike sorting), and preliminary data analysis (Fig. 1). We provide troubleshooting guidance and some alternative methods for various steps that have been developed in our lab over a decade of experiments [[2], [22], [23], [24], [25]].

## Retina preparation

### Animal preparation

To anesthetize a mouse, CO_2_ inhalation is commonly used. The anesthetizing chamber is gradually filled at a rate of 10–30% of its volume per minute. The flow of CO_2_ is continued for one minute after the mouse stops breathing. More recently, we have shifted to using isoflurane to anesthetize our mice. The primary reason is to reduce discomfort for the animal [26]. First, if the concentration of CO_2_ is increased too quickly, the gas can dissolve into the nasal mucous membranes to form carbonic acid which is a mild irritant [27]. Second, CO_2_ can produce the sensation of suffocation which is distressing to the animal. In contrast, isoflurane is understood to create only a lightheaded feeling before unconsciousness. Because isoflurane is a fast-acting anesthetic, it is understood to have minimal residual impact on the electrophysiology of neural tissue.

To assess anesthesia depth, a pinch of tissue between the mouse's toes is used to check for the pedal withdrawal reflex. This involves extending the leg and observing for an increase in muscle tension when the tissue is pinched. If a reflex is observed, the mouse is returned to the anesthesia chamber for another 60 s. Finally, the mouse is euthanized using cervical dislocation, as described in [28]. This involves placing the nonresponsive mouse on its stomach, applying firm pressure on the neck using a blunt tool like closed scissors, and pulling the tail away to dislocate the neck and crush the spinal cord. Because the cervical dislocation technique can be imperfect, especially for the inexperienced, decapitation is used to confirm euthanasia.

### Retina isolation

**Fig. 2:**
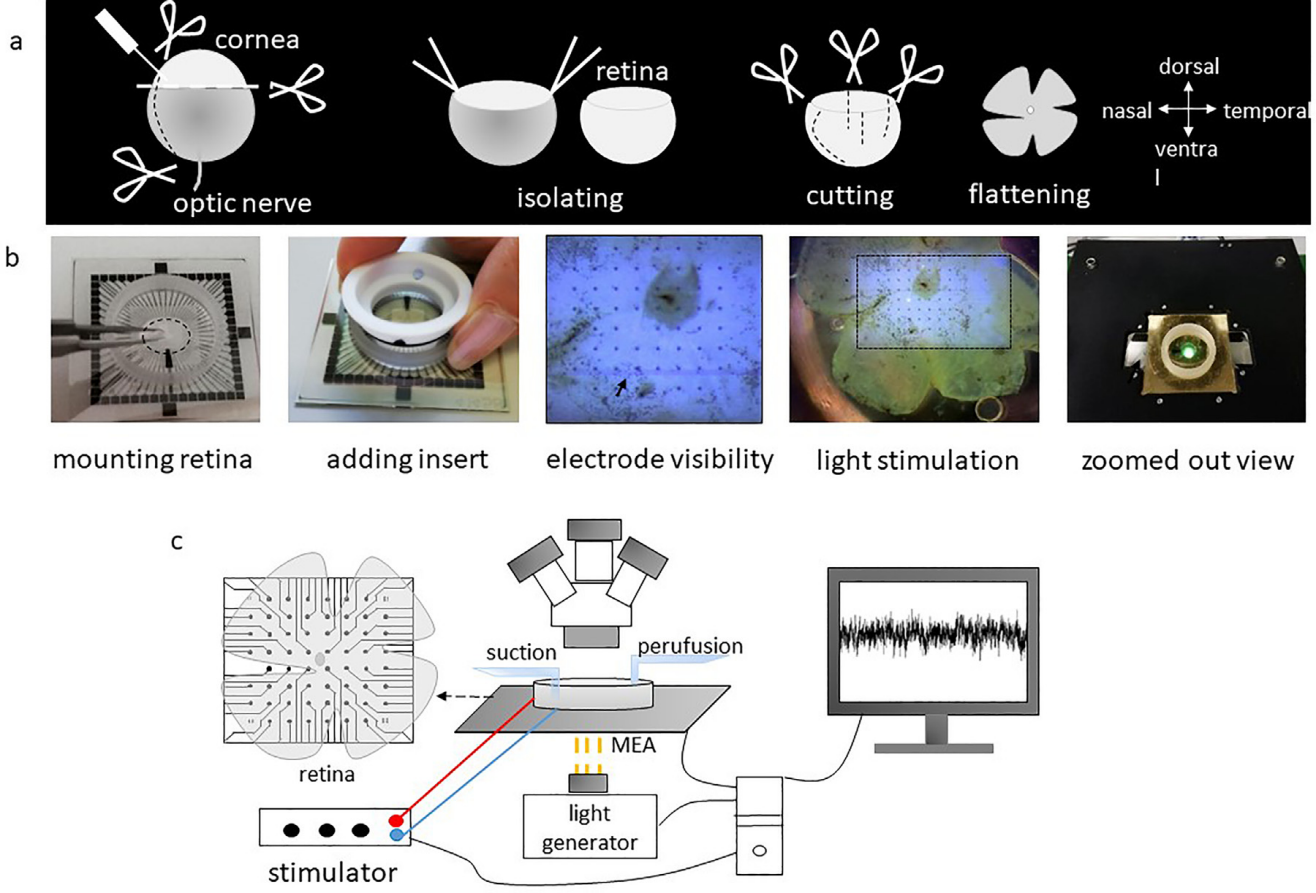
A schematic figure of the process from isolating to recording from the retina. (a) Stabbing cornea with a needle to puncture the eye and cutting the cornea from ora serrata with a pair of scissors, removing the lens and vitreous body and optic nerve, detaching retina by using two forceps and scissors, making four cuts in the retina, and finally flattening the retina on the electrodes of the MEA. (b) Images from the MEA recording setup. (c) Schematic representation of MEA recording setup: the retina is perfused with carbogenated ACSF and placed in the MEA chamber with the ganglion cell side against the electrodes. Electrical stimulation is delivered through one electrode while remaining electrodes recorded RGC action potentials extracellularly. The Light stimulus (yellow rays) is shone through the transparent MEA from below.

To track the natural orientation of the retina during surgical preparation, a cautery is used to create a small burn mark on the surface of the cornea closest to the nasal canthus, but within the corneal limbus (Fig. 2). Care must be taken to ensure that the cautery is not too hot or in contact for too long, as this could result in piercing the entire cornea and causing fluid release from the anterior chamber, making the eye more difficult to handle. To remove the eyes, gentle pressure is applied to the head to distend the eye, and curved forceps with blunt tips are opened around the eye, pressed into the socket, and gently closed around the optic nerve as the forceps are pushed under the eye. By gently pulling the eye upward, they optic nerve is torn along with muscles and connective tissue. It is important to avoid rupturing the eye during this process, as it can complicate the subsequent dissection of the retina. The removed eyes are then placed in 5 mL plastic petri dishes labeled 'Left' and 'Right', each filled with 4 mL of artificial cerebrospinal fluid (ACSF, Table S2-S4) that completely covers the eye.

The eye dissection starts by creating a hole in the cornea using a hypodermic needle inserted at the cautery point. Using Vannas scissors, the cautery mark is further extended approximately 50% of the distance to the optic disk by cutting through entire eyewall, including the retina. This creates a fiduciary mark running from the medial edge of the retina toward the optic disk. Subsequently, the cornea, ora serrata, lens, and vitreous body are removed. If not already detached, the retina is then detached from the pigment epithelium. Within the eye cup, the optic nerve is cut at the base of the retina between retina and pigment epithelium. At the point in time that the retina is separated from the rest of the eye, it is often prudent to transfer it to either the bubbler (so that the second retina can be isolated quickly) or to a dish with clean, fresh ACSF.

The final step of eye dissection is to remove any vitreous that remains on the retina. This is especially important to ensure optimal contact between the nerve fiber layer and electrodes of the MEA. Often, free floating pigment epithelium is trapped in the sticky and transparent vitreous – rendering it somewhat visible. Even when it is difficult to see, residual vitreous can be detected by closing forceps very close to the inner surface of the retina and tugging sharply. If vitreous remains, it will pull on the retinal cup and cause it to twitch. The vitreous adheres most tenaciously to the rim of the retina where the ora serrata previously was attached, and to the optic nerve head. Our preferred technique to search for and remove vitreous is to press the tips of a pair of forceps to the bottom of the dish directly in front of the retinal cup opening and insert the tips of a second set of forceps between the legs of the first set to pinch and pull away vitreous. In this way, any tugging on the retinal cup is arrested by the first set of forceps. Because the vitreous applies tension to the inner surface of the retina, the margins roll inward, causing it to often resemble a coffee bean shape. As vitreous is removed, the retinal cup will relax from this coffee bean shape to a more open bowl shape. A loose, open retinal cup - all the way out to the peripheral margins - is indicative of complete removal of the vitreous.

## Mounting retina on the MEA

### MEA chip

There are several commercially available MEAs with different electrode materials and layouts (electrode geometry, size, and spacing). Here, we use a standard MEA. This standard MEA contains 59 circular titanium nitride (TiN) electrodes (diameter: 30 µm, interelectrode spacing: 200 µm; Multi Channel Systems, Reutlingen, Germany) arrayed in an 8 × 8 rectilinear grid layout, and with indium tin oxide (ITO) electrode tracks insulated by silicon nitride (Si_3_N_4_) on a glass substrate. Electrodes were absent from the four corners of the grid and one electrode is substituted with a large reference electrode. These electrodes are sufficiently separated that individual cells are only ever recorded on one electrode at a time.

### Mounting retina on MEA

After isolating the retina from the eye, it is important to orient and flatten the retina onto the MEA using two forceps. The edges of the retina need to be unrolled or it can be cut into a square shape to establish better contact with the electrodes. We typically make three more partial cuts that cause the retina to flatten into a cloverleaf shape. These additional cuts are shorter than the fiduciary cut so that it can be easily differentiated. This maintains the whole retina and preserves its orientation, enabling us to record from any part of the retina. However, it is important to minimize cutting as it results in a damaged zone around each cut. Alternatively, using half retinas can increase data yield per animal.

### Proper contact of the retina and MEA

A dialysis membrane mounted on a custom Teflon ring is lowered onto the retina to press it against the electrodes of the MEA [16]. To keep the membrane on the Teflon ring an elastic ring is used (see design, Fig. S2). Using a new dialysis membrane for each experiment is necessary due to microbial contamination and deformation of the membrane. An alternative to the Teflon ring is treatment of the MEA surface with an adhesive substance. The MEA can be coated with Nitrocellulose, Polyethyleneimine (PEI) plus Laminin, Polyornithine (plus Laminin), Poly-d-Lysine (plus Laminin), Poly-d-Lysine (plus Fibronectin), Fibronectin, and Collagen. Additionally, harp slice grids can be used for giving good contact between tissue and MEA [the MEA Manual, https://www.multichannelsystems.com].

## Microelectrode array (MEA) recording and data acquisition

### Adjusting MEAs on the pre-amplifier

After mounting the retina, the MEA is then placed under the preamplifier (Fig. 2) and immediately superfused with carbogenated ACSF at a rate of ∼6 ml/min using a peristaltic pump perfusion system (PPS2; Multi Channel systems, Germany). The retina must receive non-stop, fresh ASCF to remain healthy. Temperature is maintained at 33 °C using both a heating plate and a heated perfusion cannula connected to a temperature controller. At the beginning of the experiment, the retina is allowed to settle and adapt to the new environment for at least 30 min before starting any data recording. This is necessary because retina preparation typically triggers a wave of spreading neural depression that takes time to resolve.

### Data acquisition and recording

For recording the spiking responses from RGCs, we use the MEA60 system (MCS, Reutlingen, Germany). In this system, data is collected using the MC_Rack program on a personal computer. The program interfaces with the MC_Card data acquisition hardware as well as an analog input card to record stimulus trigger signals.

The raw data is recorded at a rate of 50 kHz/channel with a filter bandwidth ranging from 1 Hz–3 kHz and an amplification gain of 1100. After turning off the ‘change MEA’ control within MEA_Select, one can press play in MC_Rack and see the recorded signal from each electrode. To visualize incoming data, we use a long-term Data Display, a short-term Data Display, and the unfiltered data activity for each electrode (Fig. S1). If a defective electrode is observed, it can be disconnected from its amplifier through MEA_Select. After the retina has settled and adapted for 30 min, one can press the red recording button.

## Stimulation

### Light path and optic components

**Fig. 3:**
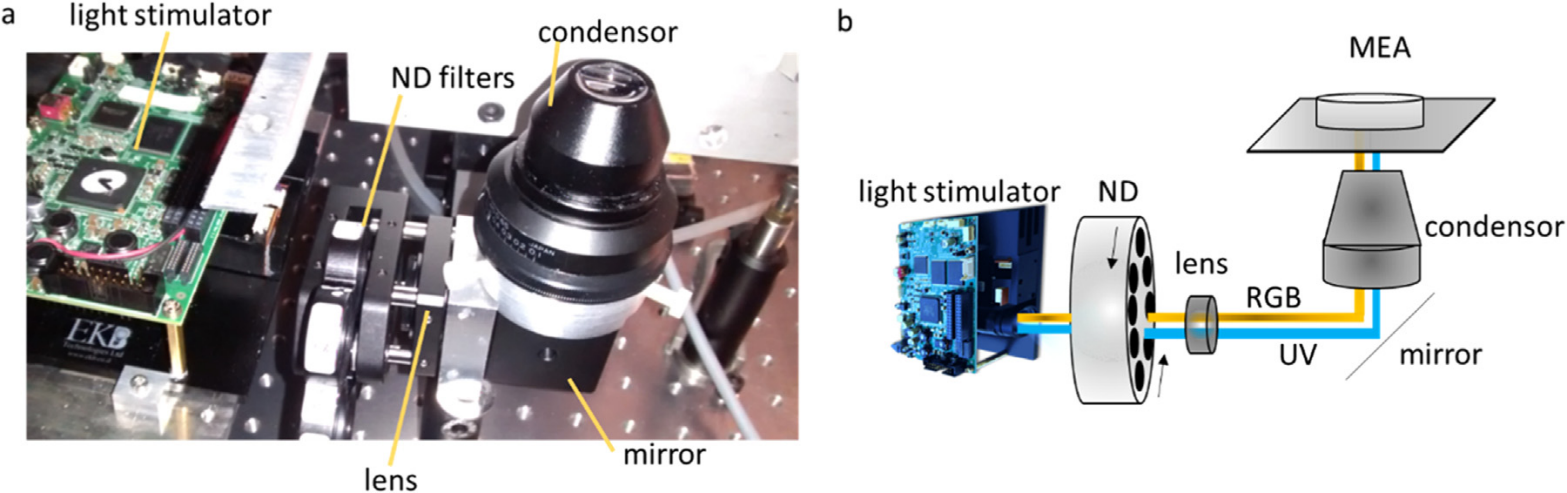
Light path of MEA system. (a) the real and (b) schematic optic path of MEA system including light stimulator, ND filters, lens, mirror, and condenser.

The light path starts with a light projector. Then the light passes through an achromatic lens doublet with a focal length of 35 mm, to a double wheel with neutral density (ND) filters of optical densities ranging from 0.1 to 8.0. This double wheel allows coarse adjustment of the overall stimulus brightness. The light passed through the ND filters is reflected by a turning mirror upward, through a condenser lens and focused onto the plane of the MEA electrodes. The whole light path is covered by a black box to reduce light reflection and scattering (Fig. 3).

### Visual stimulation

Visual stimuli are presented to the retina from below through the transparent MEA (Code_S1) with a LightCrafter 4500 patterned light generator (Texas Instruments, Dallas, USA). The LightCrafter provides a flexible light steering solution with high brightness and resolution for scientific applications. For recent experiments, the stimulator intensity (as photoisomerization rate, 10^3^ P*/s/cone) was calibrated with the white stimulus set close to 3 × 10^4^ P*/s/cone for mouse UV- and M-cones and the black stimulus was set close to 10^4^ P*/s/cone [[2], [29]]. We presented a steady mean illumination of 2 × 10^4^ P*/s/cone during the electrical stimuli and the remaining recording period to maintain the adaptation state.

### Electrical stimulation

The experimental goal for the sample data provided here is to record the responses of RGCs to voltage pulse stimulation [22]. To better understand these responses in the context of visual processing, visual stimuli are presented at regular intervals throughout the electrical stimulation protocol. The stimulus is programmed in MATLAB (The Mathworks, Natick, MA) with custom scripts (see supplement, Code_S2), and imported into the MC_Stim software. MC_Stim is used to add various timing signals to the programmed stimulus and then download it to a stimulus generator. A stimulus generator (STG 4008) is used to generate the stimulus waveforms and deliver them to the MEA. The STG 4008 enables the generation of the complex stimulus waveform - for example, biphasic, rectangular (both current and voltage). A post-time can be set in MC_Stim to facilitate discharge of the stimulating electrode. Additionally, a wait time can be set in MEA_Select to extend the blanking period before electrodes are reconnected to their amplifiers and recording resumes – thus minimizing stimulation artifacts. The MEA_Select software also allows one to select which MEA electrodes deliver the stimulus waveform.

During stimulation, the pulses are delivered from the ganglion cell side of the retina via one of the 59 electrodes – chosen based on proximity to electrodes with clear neural signals. The stimulating electrode is chosen in MEA_Select and downloaded to the blanking circuit amplifier. Prior to and after each stimulation block, spontaneous activity is recorded for 30 s as a baseline of neural activity. To monitor the stability of RGC responses over the course of the experiment, 20 cycles of 2 s ON and 2 s OFF full-field flash are presented. Thus, only the RGCs that have stable responsiveness and show no sign of rundown are included for further analysis.

### Safe charge injection for designing stimulation

MEAs have safe charge injection limit curves that show the application of maximum current and stimulus durations. Stimulus pulses should be kept safely below these limits. As a matter of fact, with high voltage or current stimulations, even below that range, check carefully to prevent faradaic reactions that will lead to electrolysis of the electrode [30]. The clearest sign of such reactions is bubble formation at the surface of either the stimulating or return electrode.

## Data processing and inclusion criteria

### Spike sorting

Accurate identification of complete and uncontaminated RGC signals is crucial prior to data analysis. RGCs generate action potentials as their output, which are recorded in the form of extracellular voltage spikes. With multiple RGCs being recorded simultaneously by a single electrode, a robust method is required to isolate individual RGC responses. This process, known as spike sorting, is essential. Here we have developed a robust semi-automated process for processing raw voltage recordings and sorting spikes into ‘units’ of similar shape which may correspond to distinct RGC sources. The best sorting of units is manually selected; and a protocol called manual cell validation is applied to determine which units can be considered as complete and uncontaminated spike trains from a single RGC. This protocol involves manually validating the responses based on various parameters to classify them as 'good' isolated RGC cell responses for further analysis or 'bad' units that are excluded from analysis. By employing this process of classification, the reliability of identifying RGC responses is enhanced. Here, we will explain automated data processing, spike sorting, and cell validation.

### Automated data processing

The stored raw data are processed using the Offline Sorter software (Plexon Inc., Dallas Tx, U.S.A.). Offline Sorter includes a batch processing feature that can read a text file and perform sorting actions based on simple commands, allowing for filtering and detection of spiking events. To process the data, voltage traces are first filtered with a 12-pole Bessel filter with a cut off frequency of 51 Hz to exclude low frequency local field potentials (LFPs) and the electrical line artifact. Following this, putative action potential events (spikes) whose filtered amplitude is greater than four standard deviations from the mean are detected.

*Sorting:* The filtered and detected events (spikes) are sorted into clusters with an automated spike sorting algorithm (T - Distribution Expectation Maximization) to assign artifact events as well as spikes from multiple cells recorded on each electrode to their own separate ‘units’. The sorting algorithm produces multiple possible sorting solutions across the specified range of the parameters.

### Cell validation

**Fig. 4:**
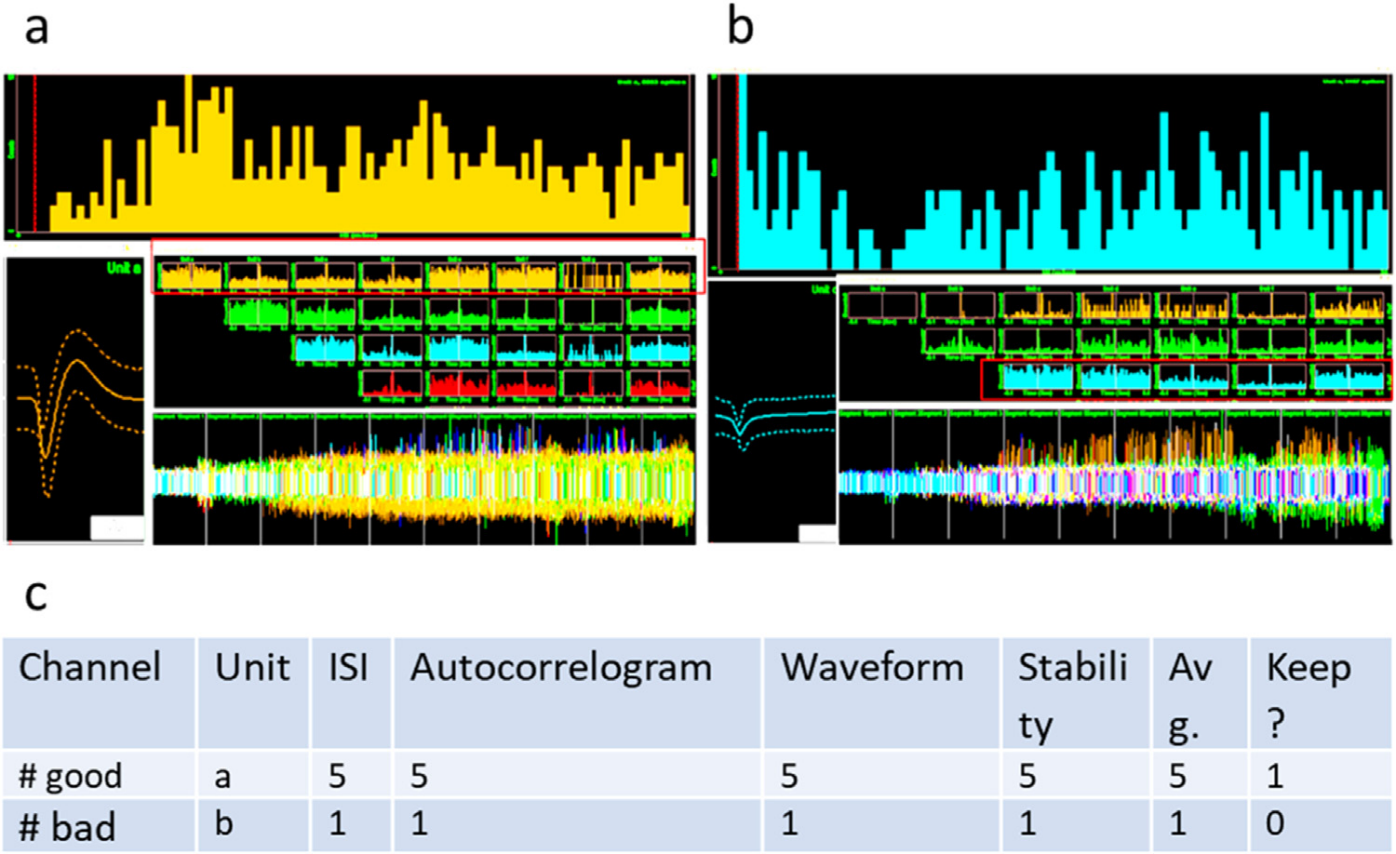
Cell validation examples. (a) Example of a ‘good’ cell. (top panel, a) ISI histogram with clear ‘lock-out’ of spikes on the far left (red vertical line indicates artificially-induced lockout due to ‘short ISI removal’ setting in Offline Sorter). (left panel, a) Waveform shape with envelope indicating variance around the mean. (middle panel, a) Pairwise cross-correlograms with the first in each row showing an auto-correlogram. (bottom panel, a) Waveform occurrences over the course of the recording. Used to evaluate waveform stability. (b) As in (a), but for a ‘bad’ example cell. (top panel, b) Note absence of a lockout in the ISI histogram, (left panel, b) a weak ‘v’ shaped waveform with no rebound, (middle panel, b) strong cross-correlation with other waveforms, and (bottom panel, b) loss of the waveform a third of the way through the experiment. (c) Table showing the cell validation for these two examples, 5 is the best score.

Cell validation is employed to ensure high confidence that the selected units represent complete spike trains of individual RGCs without contamination from other RGC spike trains or other recorded signals (Fig. 4). In cell validation, several criteria are considered including, interspike interval lock-out, cross-correlogram, waveform shape, and stability during the entire recording. These criteria are each given a score from 1 to 5 and a weighted average is calculated for each unit. With training in these methods, the scores tend to produce a bimodal distribution with a clear cutoff between bad and good units (Fig. 3).

Interspike interval lock-out: A histogram of interspike intervals is plotted. The lock-out reflects the refractory period of neuron and is an absence of events between the short-ISI removal threshold (set in Offline Sorter) and the fastest events in the histogram peak. Fig. 4a (top panel) shows a clear lock-out, whereas Fig. 4b (top panel) does not. Such events without a clear lock-out should typically be invalidated and thus excluded from analysis.

Cross-correlogram: Similar to the interspike interval histogram, the cross-correlogram plots the frequency of spikes from one unit relative to those of another unit – even with intervening spikes. The cross-correlogram between a unit and itself is an autocorrelogram. If a cross-correlogram resembles the autocorrelograms of the two contributing units, this may indicate that a single spike train has been split into multiple units (Fig. 4a, b, middle panel).

Waveform: The waveform should be negative at the first stage with a good rebound. The waveforms of cells in each cluster should be well differentiated from other waveforms. The error bars on the waveform should be small (Fig. 4a, b, left panel).

Stability: The spike shape should be consistent over the whole recording and the spike count should be reasonable for the recording time. Also, the spike frequency should be comparable across different stimuli (Fig. 4a, b, bottom panel).

## Method validation

### Visual response parameters (ON and OFF RGCs)

**Fig. 5:**
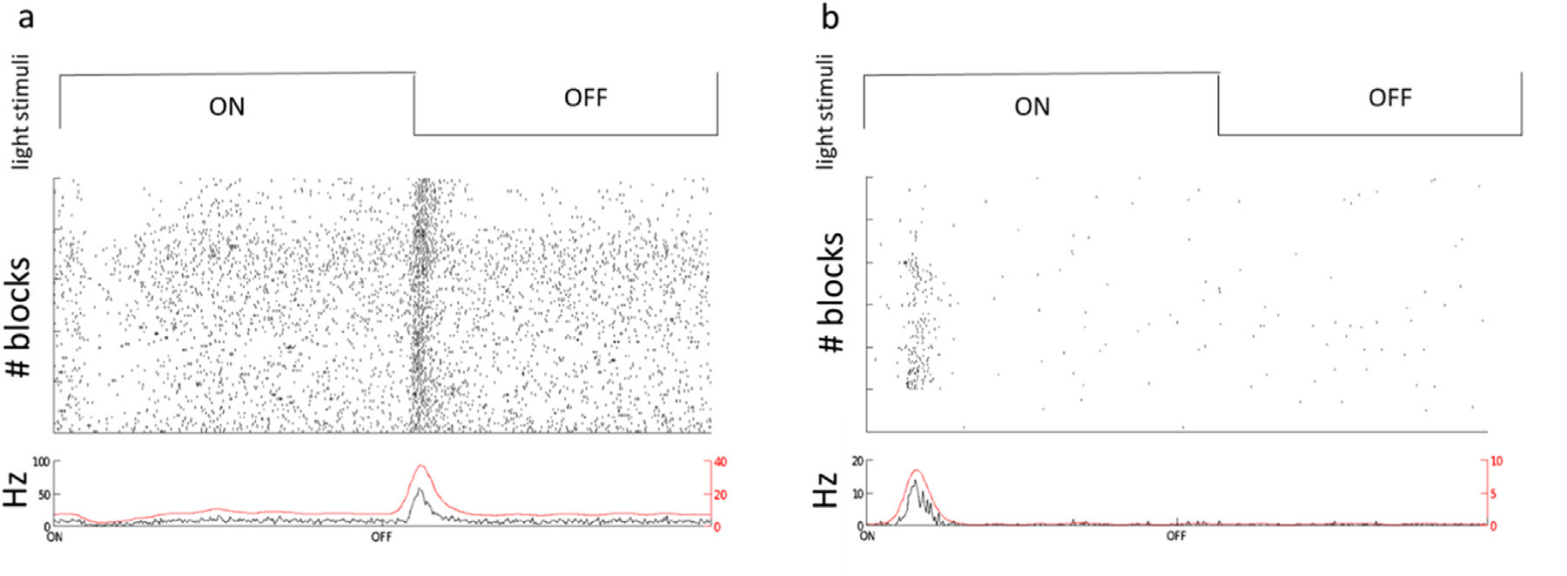
Flash responses for OFF (a) and ON (b) example RGCs. For both (a) and (b), the (top row) represents the timing of the visual stimulus of 2 s ON (∼40 klx) and 2 s OFF (∼20 lx) that is cycled 25 times per block (5 blocks). (middle row) Rastergram of spikes over the course of 5 stimulus blocks. (bottom row) Peristimulus time histogram (PSTH) aggregated across all repetitions.

For characterizing the visual responses, we used full-field flash stimuli including 2 s ON followed by 2 s OFF (Code_S1), for 100 s as shown in Fig. 5– 5 blocks). As result, in Fig. 5, we provided two examples. Fig. 5a is an ON cell and Fig. 5b is an OFF cell.We also characterized the response index across all trials based on [31] that states index of > 0.5 to 1 was an ON, < −0.5 to 1 is an OFF and an index =<0.5 and >= - 0.5 is an ON—OFF response (Code_S3) [31].

### Electrical response parameters (Voltage tuning curve of RGCs)

**Fig. 6:**
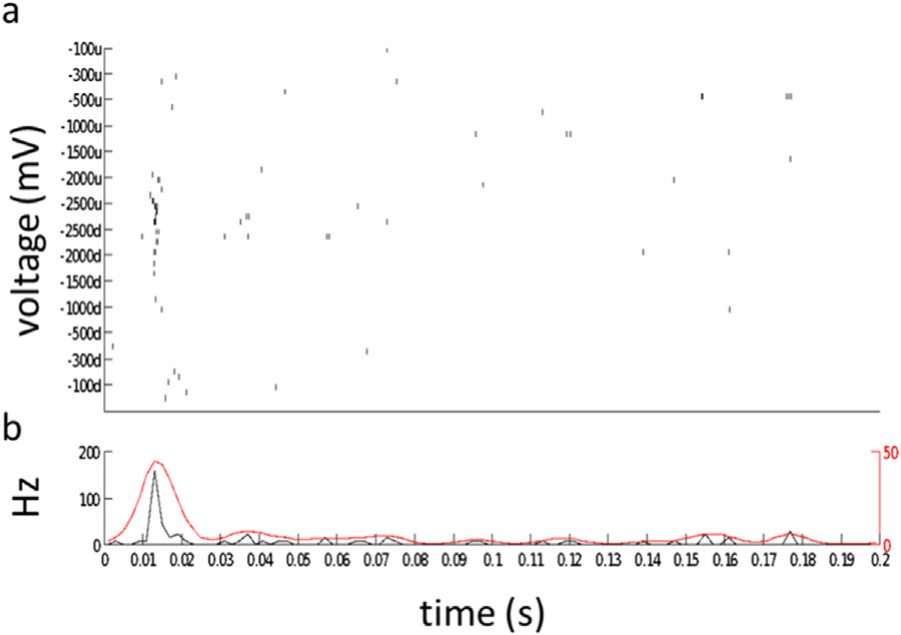
Voltage-response tuning curves for an example cell. (a) Rastergram of 5 responses to 5 presentations of each 1 ms voltage pulse. Pulses were presented in sequence first increasing (u for upward) and then decreasing (d for downward) in order to evaluate response hysteresis. (b) PSTH binned at 2 ms intervals for all responses, overlaid with a smoothed histogram (red, Gaussian smoothing filter, sigma = 4 ms).

The stimulus is delivered via an electrode of MEA consisting of monophasic cathodic rectangular voltage pulses of 1000 µs duration with the following amplitudes 100, 300, 500, 1000, 1500, 2000, and 2500 mV (Code_S2). For evaluation voltage responses to the electrical stimulus, the spiking response is integrated over the interval spanning 10 to 100 milliseconds after electrical pulse onset (Fig. 6).

The spontaneous rate assists to characterize the baseline firing rate within the context of ongoing electrical stimulation. Then threshold voltages for each cell are determined by spontaneous + 3*SD.

The response integration window of 10 to 100 milliseconds is chosen to exclude spikes generated by either direct RGC stimulation which has a latency <10 ms [17] (Code_S4). As Fig. 6 shown at higher voltage e.g., −2000 and −2500 mV stimuli elicited robust responses.

## Troubleshooting

In this section, we present essential and practical troubleshooting tips for various aspects of retinal dissection and mounting on MEAs, as well as addressing common issues related to electrical artifacts and the MEA chip setup. These troubleshooting guidelines aim to assist researchers in overcoming challenges that may arise during these critical experimental procedures, ensuring the successful acquisition of high-quality data.

### Retinal preparation and mounting on MEA

➢ Cleaning and disinfection of dissection tools: Maintaining clean and aseptic dissection tools is essential for successful recordings because it minimizes the possibility of contamination from laboratory chemicals and microbial toxins – each of which can significantly impair retinal function in vitro. After each use, it is recommended to clean the tools with distilled water and gently scrub them with a toothbrush to remove any particles or debris. Subsequently, soak the tools in ethanol (70%) for disinfection and rinse them thoroughly with distilled water to remove any residue from the ethanol.
➢ Minimizing damage during dissection: The delicate nature of retinal tissue makes it susceptible to damage during the dissection process. Care should be taken to handle the tissue gently and use precise dissection techniques to minimize any potential damage. Any damage to the retina can significantly impact its electrophysiological responses, affecting the quality of the recorded data.
➢ Challenges in Bergmeister's papilla removal: In the mouse, there is often a pigmented remnant of the hyaloid artery called Bergmeister's Papilla that may appear like pigmented epithelium suspended in vitreous near the optic nerve head. If removal of this papilla is attempted, the entire optic nerve head is often torn out – significantly damaging the retina.
➢ Prepare the artificial cerebrospinal fluid (ACSF): Stock ACSF (at 10x concentration), filtered (Merck Millipore Steritop Quick Release, Fisher Scientific) without CaCl2, MgCl2, and glucose can be stored in the fridge at 4 °C for up to 2 months (see Table S2-S4). After making 1x ACSF, glucose is added.
➢ Timing of CaCl_2_ and MgCl_2_ addition: When preparing the artificial cerebrospinal fluid (ACSF), it is important to add CaCl_2_ and MgCl_2_ after 5 min of bubbling the ACSF solution. Adding these salts before the bubbling process can cause them to precipitate out of the solution due to a high pH. By adding them at the appropriate time, their concentrations can be maintained in the desired range, ensuring optimal conditions for retinal recording. A low enough pH is reached after 5 min of bubbling to add CaCl_2_ and MgCl_2_ from already prepared stock solutions. After ∼ 15 min of carbogen bubbling, the pH equilibrates and reaches around 7.4 (Tables S9 and S10).
➢ Refreshing ACSF: It is crucial to refresh ACSF every 10 min during the dissection process. Acidification of the retina can occur rapidly, so regular refreshing of ACSF helps maintain the appropriate pH and prevents damage. In our experience, the retina is more resistant to hypoxia than to pH imbalance.
➢ Proper calcium concentration: Retinal tissue is sensitive to the concentration of calcium (Ca^+2^) due to the requirement of cell adhesion molecules in the extracellular matrix. Incorrect calcium concentration can cause retinal tissue to swell or fall apart, which can affect the overall health and viability of the tissue [32].
➢ Ensuring ACSF homogeneity for optimal recording stability: To maintain a consistent and stable extracellular recording environment as well as prevent uneven distribution of gasses and maintain a well-oxygenated ACSF solution during retinal recordings, it is important to promote the homogeneity of ACSF. This can be achieved by using a glass aeration frit connected to the O_2_/CO_2_ pipeline or employing a low bubble rate with a Pasteur pipette.
➢ Alternative recording solutions: In some cases, researchers may consider using alternative solutions for retinal recordings. One such option is Ames' solution [33].
➢ Finding correct side for mounting retina: The presence of residual pigment epithelium and the inward curvature of the retina are helpful indicators for identifying theRGC and photoreceptor sides of the retina during mounting on the MEA.
➢ Mount dialysis membrane on Teflon inserts: The dialysis membrane is carefully fixed onto the Teflon insert using an elastic ring that fits into a recessed groove. This method provides a reliable and robust attachment, preventing displacement or detachment of the dialysis membrane during the experimental procedures. Wrinkles in the dialysis membrane can deliver uneven pressure to the retina and interfere with a complete and uniform contact between retina and MEA.
➢ Teflon insert placement: Before placing the insert without disturbing the retina, ensure that all excess ACSF is removed from the chamber and the membrane. The presence of excess ACSF can cause the retina to be pulled towards the point of initial contact due to surface tension, potentially leading to displacement or damage.
➢ Avoiding electrode contact during mounting retina: Exercise caution to prevent touching the electrodes with forceps during the mounting process. The nanostructure of the electrode surface is delicate and can be damaged if handled improperly. Some investigators prefer to use ultrafine paint brushes to manipulate the retina in the MEA chamber to protect the electrodes.
  Insert with spacers: The insert used for mounting the retina should have spacers in place to prevent excessive pressure on the retina. These spacers help ensure that the retina is not compressed or damaged during the mounting process. Refer to the design and Fig. S2 for more details.

➢ Minimizing bubbles during insert mounting for optimizing electrical contact: It is important to avoid any bubbles between the dialysis membranes, retina and MEA. Bubbles around the perimeter of the retina (between membrane and MEA) should also be minimized to support optimal electrical contact.

### Electrical artifacts

➢ Controlling amplifier noise: Before running an experiment, the intrinsic level of the MEA can be tested by filling it with an electrolyte solution (saline or PBS; Table S1). Amplifier noise should be around ±8 µV for a standard MEA (for further information see the MEA Manual; https://www.multichannelsystems.com).
➢ Photovoltaic response at high light levels: At very high light levels, electrodes can generate a photovoltaic response in the absence of tissue. However, this response may be attenuated in the presence of tissue. To ensure accurate measurements and minimize potential artifacts, it is advised to avoid shining high-intensity light during the process of controlling amplifier noise.
➢ Artifact and baseline variation: The size of the electrode's diameter can influence the baseline observed during recordings. Generally, smaller electrode diameters tend to exhibit a larger baseline variation. It is important to note that baseline artifacts with tissue on the MEA are typically lower compared to recordings without tissue.
➢ Baseline voltage oscillations from perfusion pulsations: Oscillations of the baseline recording voltage can result from perfusion pulsations. In this case, the oscillations match the pump in frequency and go away when the pump is turned off. A pulse dampener (e.g., the ‘droplet isolator chamber’ cited in user manual, part: PPS2-Set-F, https://www.multichannelsystems.com) reduces these oscillations [34]. Make sure the dampener is hanging vertically and reintroduce a bubble of air to the dampener. Unlike the ACSF, the air compresses with each pulse and this compressed air provides steady pressure to the outflow between pulses.
➢ Spiky artifacts: If spiky artifacts are observed across multiple electrodes and are highly correlated, it may indicate the presence of bubbles in the perfusion system. To address this issue, try switching the perfusion on and off while allowing the suction to run dry. This process can help remove any potential bubbles that might be causing the artifacts.
➢ Irregular sharp artifacts caused by bubbles: If irregularly timed, sharp artifacts persist on all electrodes and are unaffected by turning off the suction, it may indicate the presence of bubbles in the chamber. To resolve this issue, ensure that the perfusion intake is positioned away from the bubbler and check for bubble formation in the perfusion line or chamber. Additionally, minimizing the cooling of the ACSF from the perfusion reservoir to the chamber can help prevent the release of carbogen and reduce bubble formation.
➢ Artifact from perfusion droplets: The perfusion droplets are another source of artifact which can be resolved with a pulse-dampener. The perfusion tip should be cut parallel to the surface of the ACSF and should share a liquid meniscus with the chamber so that individual droplets do not lead to artifacts. To ensure that perfusion and suction occur at even rates, the tip of the suction line meets the liquid at an acute angle and is cut perpendicular to the liquid surface. The suction pump is run at a higher flow rate than the perfusion pump and is always ‘slurping’ a combination of air and ACSF. These details ensure a self-regulating system based on the triangular surface area of the suction tip. If the fluid level rises, the submerged area of the triangle and the corresponding rate of liquid removal increases; if the fluid level drops, the submerged area and the rate of liquid removal decreases. This system is commonly used in patch-clamp experiments.
➢ Artifact from contaminated contact pads or amplifier pins: To avoid poor recording quality or noisy signals, it is recommended to carefully clean the MEA contact pads and preamplifier contact pins with ethanol on a cotton swab each time before the preamplifier is placed on the MEA. This removes any debris or contaminants like skin oils that may interfere with signal transmission. Also, inspect the pins to ensure they are not bent, broken, or stuck.
➢ Artifact from loose mechanical contact: It is possible that loose mechanical contact between the preamplifier contact pins and the MEA contact pads can produce an artifact. This artifact can be eliminated by tightening the screws of the two white stripes on the sides of the preamplifier.
➢ Artifact on a single electrode: artifacts on a single electrode could result from the electrode's pin on top of the preamplifier being in contact with an unintended object or surface. Check for any physical contact between the electrode's pin and surrounding elements, ensuring that it is properly positioned without interference.

### MEA chip and setup

➢ Cleaning MEA for experiment: Clean MEA before experiment. The MEA is immersed in a Tergazyme solution (5 mM, Alconox,Inc.) for 3 hours or over night. Afterward, carefully clean it with ethanol (70%) and rinse it thoroughly with distilled water at least five times before starting an experiment.
➢ Monitoring electrode impedances for recording quality control: Before and after an experiment the impedances of the electrodes in ACSF is measured at 1 kHz by using a NanoZ impedance tester (Multichannel Systems., Reutlingen, Germany).
➢ Proper cleaning of the perfusion path: After completing an experiment, it is crucial to clean the perfusion path to maintain its functionality and prevent any residual buildup. Begin by flushing the path with distilled water to dilute any remaining ACSF. Subsequently, flush the path with 70% ethanol at a flow rate of 4 mL/min for 30 min to disinfect and remove any organic contaminants. Proper cleaning of the perfusion path helps maintain a clean and uncontaminated environment for future experiments. Because microbes – especially fungi – can grow in the smallest droplets of water, it is recommended to use compressed air to blow out the perfusion lines and dry them as much as possible.
➢ Periodic descaling of the perfusion path: To prevent salt sedimentation and ensure optimal perfusion, it is important to descale the perfusion path on a regular basis. Weekly descaling is recommended to remove any accumulated accumulation. Start by flushing the path with distilled water until it runs dry, followed by flushing with citric acid solution (1 M) at a flow rate of 4 mL/min for 30 min to decalcify and remove sedimentation. Finish by thoroughly flushing the path with distilled water multiple times.
➢ Ensuring proper ACSF flow and tubing alignment: Prior to mounting the retina on the MEA, it is important to run ACSF through the tubing. This step ensures that the perfusion and suction systems are functioning and that the retina receives ACSF immediately upon placement in the recording rig. Running ACSF through the tubing also helps identify any potential leaks or misalignments in the tubing and tips.
➢ Tubing volume and duration for optimal temperature and gas solution in setup: Thinner tubes tend to lose temperature more rapidly, while some types of tubing may be permeable to gasses, leading to the loss of carbogen. Minimizing the length of connector tubes helps preserve temperature, gas solution, and pH stability during the recording.
➢ Optimizing amplifier recovery with change MEA mode: The Change MEA button sets all electrodes to the ground and is used for manipulations on the MEA amplifier. Missing the activation of the Change MEA mode before lifting the preamplifier off the MEA can lead to a very long time until the amplifier has recovered and is ready for operation due to saturation (several seconds to minutes).
➢ Verifying data and power connections: Before commencing the experiment, it is crucial to double-check all data and power connections. Ensure that all cables and connectors are securely plugged in and properly seated. This step helps prevent any potential interruptions or data loss during the recording session.

Why are there no spikes?

➢ In establishing a new MEA system or new experimentalist, we have often encountered the problem of not being able to record neural signals on the MEA – despite an apparently good retinal preparation. There are many potential causes for this that should each be eliminated. Our main advice is to be persistent in performing regular experiments – despite continued failure – until successful recordings are made. Then maintain the techniques that resulted in good recordings. We have found that the most likely candidates for missing spikes are poor tissue-electrode contact – especially from residual vitreous, microbial contamination, chemical contamination, ionic imbalance, or pH imbalance.
➢ To troubleshoot for residual vitreous, we recommend performing a few ‘extreme’ dissections where the experimenter is excessively diligent in vitreous removal techniques to see if this solves the problem.
➢ In the case of poor contact, despite rigorous vitreous removal, it can be useful to position parts of the retina with variable thickness over the electrode array to evaluate whether tissue contact is too light or too heavy. This can include the edge of the retina which is tapers in thinness, tears and cuts in the retina, and unintentional folds in the retina.
➢ Microbial contamination can be insidious because, even with some sterilization techniques, microbial growth that has been sterilized to kill the microbes can leave behind toxic metabolites that are not destroyed by the sterilization technique. Cleaning with citric acid and immediate flushing with de-ionized water followed by thorough drying should resolve this problem.
➢ Similarly, many chemicals used in a histology lab (e.g., paraformaldehyde) can leave behind residue that is toxic to the sensitive retina. We recommend buying new glassware and retaining it only for MEA experiments. Additionally, neurotoxins often used in MEA experiments can persist in the perfusion system. The best practice is to replace all tubing and connectors and thoroughly clean the system with citric acid.
➢ If you mix your own ACSF, it is possible to improperly measure the components. In particular, a 2-fold mistake in either Ca^2+^ or Mg^2+^ concentrations can eliminate neural signals. If Ca^2+^ concentration is too low, the extracellular matrix of the retina may also break down – turning the retina into a mush of cells that cannot be handled.
➢ The mouse retina appears to be particularly sensitive to acidosis. We have found that more than 10 min without maintenance of physiological pH is sufficient to eliminate neural responses. Interestingly, mouse retina readily recovers from up to 30 min of low oxygen.
➢ We have not found that room temperatures are cold enough to shut down neural activity. Temperatures around 17 °C tend to merely reduce the frequency of action potentials and diminish the robustness of spiking responses to stimuli. The retina also readily recovers when physiological temperature is restored.

## Ethics statements

The experiments described here complied with the ARRIVE guidelines and were carried out in accordance with the National Institutes of Health guide for the care and use of laboratory animals (NIH Publications No. 8023, revised 1978). All experimental procedures have approval of the state authorities (Regierungspraesidium, Tuebingen) and were conducted under the supervision of the Tuebingen University facility for animal welfare (Einrichtung fuer Tierschutz, Tieraerztlichen Dienst und Labortierkunde).

## Funding

This work was supported by the 10.13039/501100001659Deutsche Forschungsgemeinschaft (HO 6221/1–1) and ERC (101039764 – NeuFRO**)** to Z.H., by the Bundesministerium für Bildung und Forschung (031a308) to D.L.R., by PRO RETINA (Pro-*Re*/KP/Hosseinzadeh.1–2020) to Z.H., and by the Tistou and Charlotte Kerstan Foundation to Z.H.

## CRediT authorship contribution statement

**D.L. Rathbun:** Conceptualization, Methodology, Validation, Resources, Writing – review & editing, Supervision, Project administration, Funding acquisition. **A. Jalligampala:** Methodology, Validation, Investigation, Writing – review & editing. **E. Zrenner:** Supervision, Resources, Funding acquisition. **Z. Hosseinzadeh:** Conceptualization, Methodology, Validation, Resources, Writing – original draft, Writing – review & editing, Supervision, Funding acquisition.

## Declaration of Competing Interest

The authors declare that they have no known competing financial interests or personal relationships that could have appeared to influence the work reported in this paper.

## Data Availability

All data and codes are in the supplement.

## Appendix Supplementary materials

Image, application 1
