# Supplementary material for: Improvements for recording retinal function with Microelectrode Arrays
Source: MethodsX. 2023 Dec 29;12:102543. doi: 10.1016/j.mex.2023.102543 (PMC10834997; doi:10.1016/j.mex.2023.102543)
Supplement: Supplementary file 1 [file mmc1.docx]

# Supplement information

# Article title

## Improvements for recording retinal function with Microelectrode Arrays

# Authors:

D.L. Rathbun *^,+, 1^, A. Jalligampala^+2^, E. Zrenner^3,4^ , ^5^Z. Hosseinzadeh ^*,5^

**Supplementary tables**

**Table S1:** PBS Buffer

ddH2O 2L

NaH_2_PO x H_2_O 5,52g

Na_2_HPO_4_ x 2H_2_O 28,48g

NaCl 18g

pH 7,4

**Table S2:** ACSF Stock (10x)

ddH_2_O 500mL

NaCl 36,53g 1250 mM

NaHCO_3_ 10,50g 260 mM

NaH_2_PO_4_ 0,75g 12.5 mM

KCl 1,31g 35 mM

**Table S3:** Stock Salts (1000x)

ddH_2_O 50 mL

MgCl_2_ 10.17 g 1 M

ddH_2_O 50 mL

CaCl_2_ 14.70 g 2 M

**Table S4:** ACSF (1x)

ddH_2_O 900mL

ACSF (10x) 100mL

MgCl_2_ (1000x) 1mL 1 mM

CaCl_2_ (1000x) 1mL 2 mM

Glucose 4.504 g 25 mM

**Table S5:** List of chemicals

| **Name** | **Company** | **Catalog Number** | **Comments** |
| --- | --- | --- | --- |
| Citric acid) (HOOCCH₂)₂C(OH)COOH | Merck Millipore | 100241 | 5 kg  224,00€ |
| Magnesium chloride hexahydrate (MgCl_2_ x 6H­_2_O) | Sigma-Aldrich | M2670 | 100g  46,40€ |
| Sodium chloride (NaCl) | Sigma-Aldrich | S7653 | 1kg  93,50€ |
| Calcium chloride dihydrate (CaCl_2_ x 2H_2_O) | Sigma-Aldrich | C7902 | 100g  102,00€ |
| Potassium chloride (KCl) | Sigma-Aldrich | P9333 | 500g  73,90€ |
| Sodium bicarbonate (NaHCO_3_) | Sigma-Aldrich | S5761 | 1kg  72,80€ |
| Sodium phosphate monobasic (NaH_2_PO_4_) | Sigma Aldrich | S8282 | 500g  171,50€ |
| D-(+)-Glucose (C_6_H_12_O_6_) | Sigma-Aldrich | G8270 | 1kg  44,50€ |
| Terg-a-zyme | Sigma-Aldrich | Z273287-1EA | 1EA  45,90€ |

**Table S6:** List of instruments

| **Name** | **Company** | **Catalog Number** | **Comments** |
| --- | --- | --- | --- |
| HE-Inv-8 | Multi Channel Systems MCS GmbH |  | Heating element |
| PH01 | Multi Channel Systems MCS GmbH |  | Heatable perfusion canula |
| MEA1060-Inv-BC | Multi Channel Systems MCS GmbH |  | Amplifier for 60 electrodes |
| Stimulation generator  STG4008 or STG2008 | Multi Channel Systems MCS GmbH |  | Stimulus generator |
| DCC 1645C - High Resolution Camera | Thor labs | DCC1645C | 319,50€ |
| Preamplifier | Multi Channel Systems | MEA1060-Inv-BC |  |
| RS-232 | Multi Channel Systems |  | Serial port |
| Amplifier | Multi Channel Systems | FA605BC |  |
| MC card | Multi Channel Systems | CH1-64 |  |
| Light Crafter | EKB | DLP 4500 |  |
| Steritop Filter | Merck Millipore | SCGPT02RE | 95,90€ |

**Table S7:** List of software

| **Name** | **Company** | **Comments** |
| --- | --- | --- |
| Offline Sorter | Plexon Inc. Dallas, Texas, USA | http://www.plexon.com |
| NeuroExplorer | Plexon Inc. Dallas, Texas, USA | http://www.plexon.com |
| MEA select 1.3.0 | Multi Channel Systems MCS GmbH | Software |
| MC stimulus | Multi Channel Systems MCS GmbH | STG-software |
| PPS2 | Multi Channel Systems MCS GmbH | Peristaltic Perfusion System Software |
| Nano Z 1.4 | Multi Channel Systems MCS GmbH | Impedance reader Software |
| TCX-Control setup 1.3.4 | Multi Channel Systems MCS GmbH | Software |
| Sync Toy 2.1 | Online free software | Synchronizing software |
| MATLAB (the Mathworks, Natic, MA) | [https://de.mathworks.com](https://de.mathworks.com/) | Program language |

**Table S8**: List of tools

| **Name** | **Company** | **Catalog Number** | **Comments** |
| --- | --- | --- | --- |
| Tweezer Dumont #5 | W.P.I. | 500233 | $64 |
| Tweezer Dumont #7 | W.P.I. | 14097 | $44 |
| Vannas Scissors | W.P.I. | 14122 | $284 |
| Instrument storage Portfolio | W.P.I. | 503294 | $39 |
| Cautery Kit | F.S.T. | 18010-00 | 67,00€ |
| Da Vinci Pinsel Serie15872 | NeoLab | 372620000 | 3,24€ |
| glass Aeration Frit | Hugo Sachs Elektronik  (Harvard Apparatus) | T28004 | 21,6€ |

**Table S9:** The PH measurement from turning on carbogen

| Time after running carbogen | PH |
| --- | --- |
| 5 min | 7.55 |
| 6 min | 7.53 |
| 8 min | 7.49 |
| 10 min | 7.46 |
| 15 min | 7.43 |

**Table S10:** The PH measurement from turning off carbogen

| Time after carbogen off | Time |
| --- | --- |
| 2min | 7.52 |
| 3 min | 7.52 |
| 7 min | 7.53 |
| 10 min | 7.54 |
| 20 min | 7.56 |

**Supplementary Figure**


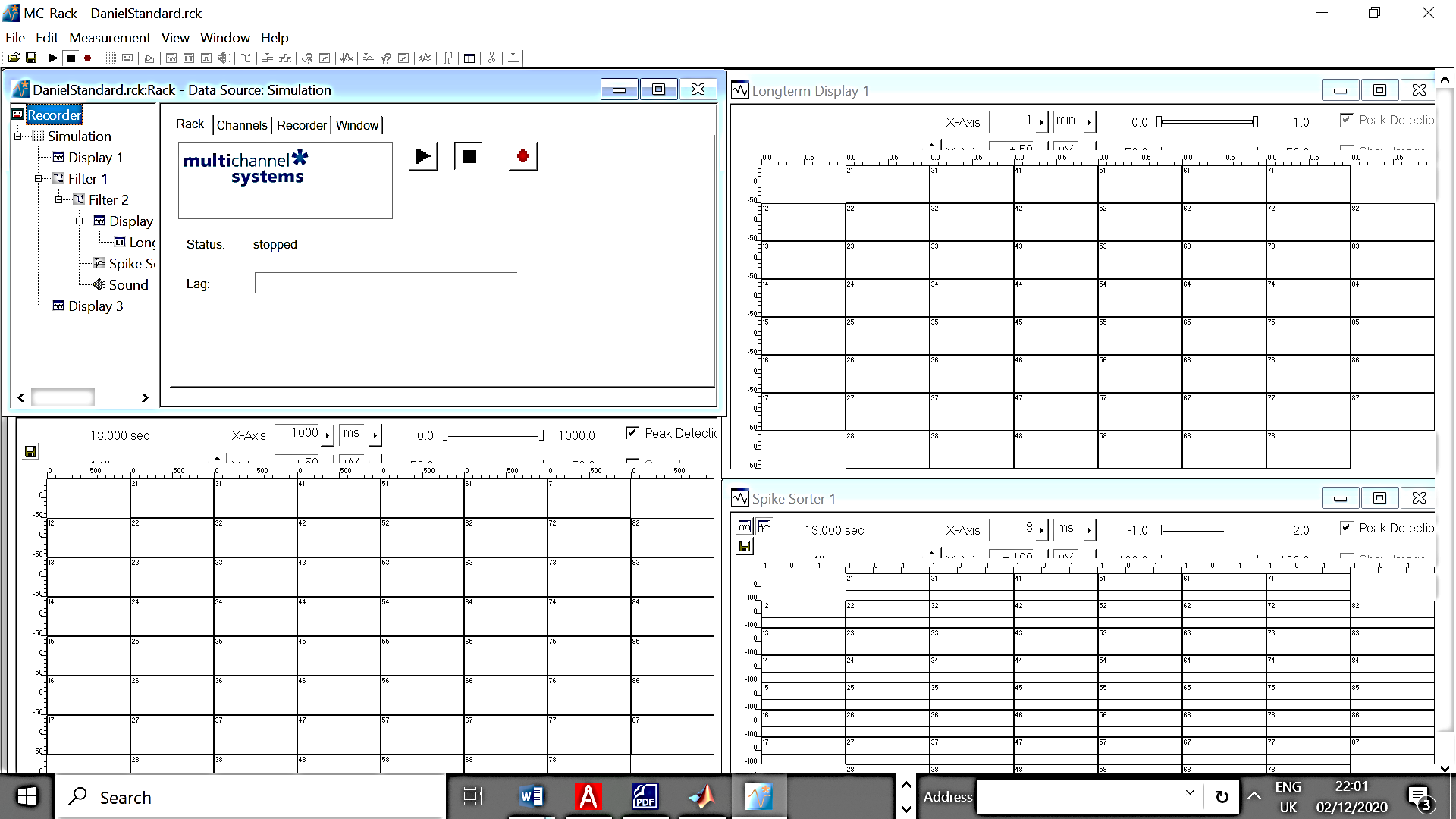


**Fig S1:** MC Rack recoding windows including long and short displays as well as spike sorter.

Fig. S2: A diagram of the Teflon insert. Different measurements of different dimensions: a) From the top, noting diameter measurements. b) and c). Cross-section of the insert. d) 3D rendering of the insert. All dimensions in mm.

**Custom codes**

Code_S1_ Visual stimulus (from Thomas Euler ‘ lab, see: )

import QDS

#import math

QDS.Initialize("ERP_RGC_on_off_2s_3", "Full field flash 2s on 2s off x20")

p = {"nTrials" : 20,

"tSteadyOFFs" : 2.0, # Light OFF

"tSteadyON_s" : 2.0, # Light 100% ON

"RGB_IFull" : (0,154,92),

"RGB_IFull1" : (0,0,0),

"dxStim_um" : 5000, # Stimulus size

"StimType" : 1, # 1 = Box, 2 = Circle/

"durFr_s" : 1/60.0, # Frame duration

"nFrPerMarker" : 3}

QDS.LogUserParameters(p)

durMarker_s = p["durFr_s"]*p["nFrPerMarker"]

#RGB_IFull = 3 *(p["IFull"],)

#RGB_IFull1 = 3 *(p["ILow"],)

QDS.DefObj_Box(1, p["dxStim_um"], p["dxStim_um"], 0)

QDS.DefObj_Ellipse(2, p["dxStim_um"], p["dxStim_um"])

QDS.StartScript()

for trg in range(10):

QDS.Scene_Clear(durMarker_s, 0)

QDS.Scene_Clear(durMarker_s, 1)

QDS.Scene_Clear(durMarker_s, 0)

QDS.Scene_Clear(.5, 1)

QDS.Scene_Clear(durMarker_s, 0)

#QDS.Scene_Clear(p["tSteadyOFFs"] -durMarker_s, 0)

for iL in range(p["nTrials"]):

#QDS.Scene_Clear(durMarker_s, 0)

#QDS.Scene_Clear(p["tSteadyOFFs"] -durMarker_s, 0)

#QDS.SetObjColor(1, [p["StimType"]], [RGB_IFull])

QDS.SetObjColor(1, [p["StimType"]], [p["RGB_IFull"]])

QDS.Scene_Render(p["tSteadyON_s"], 1, [p["StimType"]], [(0,0)], 1)

#QDS.SetObjColor(1, [p["StimType"]], [RGB_IFull1])

QDS.SetObjColor(1, [p["StimType"]], [p["RGB_IFull1"]])

QDS.Scene_Render(p["tSteadyOFFs"], 1, [p["StimType"]], [(0,0)], 0)

# QDS.Scene_Clear(durMarker_s, 1)

# QDS.SetObjColor(1, [p["StimType"]], [RGB_IFull])

# QDS.Scene_Render(p["tSteadyON_s"], 1, [p["StimType"]], [(0,0)], 0)

#

# QDS.SetObjColor(1, [p["StimType"]], [RGB_IFull1])

# QDS.Scene_Render(p["tSteadyOFFs"], 1, [p["StimType"]], [(0,0)], 0)

#

# QDS.Scene_Clear(durMarker_s, 1)

#

QDS.Scene_Clear(durMarker_s, 0)

# QDS.Scene_Clear(p["tSteadyOFFs"] -durMarker_s, 1)

# QDS.Scene_Clear(durMarker_s, 1)

# QDS.Scene_Clear(p["tSteadyOFFs"] -durMarker_s, 0)

QDS.SetBkgColor((0,104,62))

QDS.EndScript()

# -----------------------------

Code_S2_ Electrical stimuli

close all;

clear; clc

Protocol = {'UpDown'; 'DownUp'; 'Random'}; %three different recall

condition = 1; % 1:'UpDown'; 2='DownUp'; 3:'Random'

duration = 1000; % (µs) duration of each puls

Interval = 4999000; %Interval duration (microseconds)

Voltage = [-100, -300, -500, -1000, -1500, -2000, -2500 ]; % introdunce voltages

Reps = 5; %Repetitions

FileName = strcat('Volts_',Protocol(condition));

Header = {'Multi Channel Systems MC_Stimulus','ASCII import Version 1.10',...

'channels: 8','output mode: voltage','format: ','1','channel: 1',...

'value','time','value','time'};

path = 'D:\Projects_Zohreh\Electrical_Stimulation';

if ~exist(path)

mkdir(path);

end

fid=fopen(char(strcat(path, '\', FileName, '.dat')),'w');

fprintf(fid,'%s\r\n%s\r\n%s\r\n%s\r\n%s\t%s\r\n\r\n%s\r\n%s\t%s\t%s\t%s\r\n',Header{:});

for i1=1:Reps

for i2=1:length(Voltage);

Stim={num2str(Voltage(i2)) ,num2str(duration), num2str(0),num2str(Interval)};

fprintf(fid,'%s\t%s\t%s\t%s\r\n',Stim{:});

end

for i2=length(Voltage):-1:1;

Stim={num2str(Voltage(i2)) ,num2str(duration), num2str(0),num2str(Interval)};

fprintf(fid,'%s\t%s\t%s\t%s\r\n',Stim{:});

end

end

Code_S3_ Characterisation of ON and OFF RGCs

%% Response characterization per Carcieri et al. 2003

for i1=1:(length(Data)) %Cells

PSTH=mean(Data(i1).hst,2)/binw; %convert the histogram to Hz

SmFilt=pdf('norm',-filtw*3:filtw*3,0,filtw); %create the smoothing filter

PSTH=conv([PSTH(end-filtw*3:end-1);PSTH(1:end-1);PSTH(1:filtw*3)],SmFilt,'valid'); %Assuming it's cyclic, we can pad the ends. [fix this for non-cyclic data]

if ~isempty(PSTH) %Catch cells with no spikes during stimulation.

%Calculate baseline values

if length(BLwin)==2

elseif length (BLwin)==4

bline=mean(PSTH([1+BLwin(1)/binw:BLwin(2)/binw,1+BLwin(3)/binw:BLwin(4)/binw])); %Calculate Baseline (Hz)

sig=std(PSTH([1+BLwin(1)/binw:BLwin(2)/binw,1+BLwin(3)/binw:BLwin(4)/binw])); %Calculate the standard deviation of Baseline (Hz).

end

%ON response

[c,lat(1)]=max(PSTH(1:xtk(2)/binw)); %Find peak latency

amp(1)=PSTH(lat(1))-bline; %Peak amplitude (Hz)

% if amp(1) > 2*sig %Catch responses lower than 2SD over baseline.

dur(1)=min([inf (find(PSTH(lat(1):xtk(2)/binw) <= bline+amp(1)/exp(1) , 1)-1)]); %duration is the earliest response after peak <= pk*.3679

% else

% amp(1)=0; dur(1)=0; lat(1)=0;

% end

%OFF response

[c,lat(2)]=max(PSTH(1+xtk(2)/binw:xtk(3)/binw)); %Find peak latency

amp(2)=PSTH(lat(2)+xtk(2)/binw)-bline; %Peak amplitude (Hz)

% if amp(2) > 2*sig %Catch responses lower than 2SD over baseline.

dur(2)=min([inf (find(PSTH(lat(2)+xtk(2)/binw:xtk(3)/binw) <= bline+amp(2)/exp(1) , 1)-1)]); %duration is the earliest response after peak <= pk*.3679

% else

% amp(2)=0; dur(2)=0; lat(2)=0;

% end

Data(i1).bline=bline; Data(i1).sig=sig; Data(i1).lat=lat*binw;

Data(i1).amp=amp; Data(i1).dur=dur*binw; %convert to seconds

end

end

save([fpath strrep(fname,'.mat','_FlashData.mat')],'Data');

Code_S4_ Voltage Tuning curve analysis

function [Data]=ZohrAna1(fname,jnk,StmLst,trgs,xlim,binw,xtk,xlbl,filtw) %ZOHReh ANAlysis program #1

% function [Data]=ZohrAna1(fname,jnk,StmLst,trgs,xlim,binw,xtk,xlbl,filtw)

% [Data]=ZohrAna1('E:\specialextent13-11-15\spatial-right-p-13-11-15\spatial-right-p-13-11-15.mat',...

% 2,{'-100u','-300u','-500u','-1000u','-1500u','-2000u','-2500u',...

% '-2500d','-2000d','-1500d','-1000d','-500d','-300d','-100d'},'trgs=RawDat.A2a(70:end);',xlim,binw,xtk,xlbl,filtw);

% Runs initial analysis on Zohreh's MEA data to produce rastergrams and a

% cumulative PSTH for each unit.

% fname=string containing workspace file name with full directory location.

% jnk=number of trigger files (junk) in the workspace.

% StmLst=cell array containing the stimulus block labels (strings) in the

% order they were presented - for labeling the Y axis of the rastergram.

% trgs=string argument that will be evaluated to assign the trigger times.

% xlim,binw,xtk,xlbl,filtw - for rPSTH see rPSTH help files

% Data=a standardized data structure that can be used for subsequent

% analysis. Contains:

% name = unit name

% spks = list of spike times for each unit

% trgs = list of trigger times for the whole recording

% ytk = y axis tick list delineating stimulus (voltage) blocks

% StmLst = list of stimulus voltages (y tick labels) in order of presentation.

% Requires the associated RandDur stimulus design .DAT files.

% Called by ZohRerun.m

% Calls rPSTH.m saveppt2.m

%Adapted by Daniel L. Rathbun from ArchAna1.m 20160104

%REVISIONS

RawDat=load(fname); %load the workspace

RawDataFieldNames= fieldnames(RawDat);

vnames=sort(RawDataFieldNames); %get unit names

slashind=strfind(fname,'\');

expname=fname(slashind(end)+1:end-4); %get the filename

eval(trgs); %assign the triggers

%collect the stimulus values in the order they were presented.

bob=importdata('.dat','\t',8);

Volts = bob.data(:,1); %The order of voltage presentations in one stimulus block.

% Re-order the stimuli for rastergram plotting

TClen=length(unique(Volts))*2; %The length of one full up/down stimulus cycle.

BlockReps=length(trgs)/length(Volts); %Stimulus block repetitions

ind=1; TCBlockVolts=[]; TCBlockTrgs=[];

for i1=1:TClen %each value in one up/down stimulus cycle.

for i2=1:BlockReps %each stimulus block

for i3=i1:TClen:length(Volts) % skip through identical points in each stimulus cycle of one stimulus block.

TCBlockVolts(ind)=Volts(i3);

TCBlockTrgs(ind)=trgs((i2-1)*length(Volts)+i3);

ind=ind+1;

end

end

end

%assign the unit names and spike times to a data structure for output.

% for i1=1:length(vnames)-jnk %size(RawDat.Data, 1)

% Data(i1-jnk).name=vnames(i1-jnk);

% eval(['Data(i1-jnk).spks=RawDat.',vnames{i1-jnk},';']);

% end

for i1=1:length(RawDat.Data)

Data(i1).name = RawDat.Data(i1).name;

Data(i1).spks = RawDat.Data(i1).spks;

end

Data(1).Volts=TCBlockVolts;

trgs=TCBlockTrgs';

Data(1).trgs=trgs;

trgs=[trgs+xlim(1),trgs+xlim(2)]; %add end times for response binning and plotting

ytk=[];

for i1=1:length(TCBlockVolts)/(BlockReps*length(Volts)/TClen); ytk(i1)=(BlockReps*length(Volts)/TClen); end %get the number of presentations of each unique stimulus.

ytk=ytk(end:-1:1); %reverse the order for rastergram plotting.

for i1=2:length(ytk); ytk(i1)=ytk(i1)+ytk(i1-1); end %convert block lengths to cumulative values.

% initialize the powerpoint (see saveppt2 help).

pptName=strrep(fname,'.mat','.ppt');

ppo=saveppt2(pptName,'init','driver','meta','visible','on');

saveppt2(pptName,'ppt',ppo,'figure',0,'title',expname);

%run rPSTH for each unit.

for i1=1:(length(Data))

if nargin>8

[fh,hst,PSTH]=rPSTH(Data(i1).spks,trgs,xlim,binw,xtk,xlbl,ytk,StmLst(end:-1:1),Data(i1).name,filtw);

else

[fh,hst,PSTH]=rPSTH(Data(i1).spks,trgs,xlim,binw,xtk,xlbl,ytk,StmLst(end:-1:1),Data(i1).name);

end

% [fh,hst,PSTH]=rPSTH(spikes,trgs,xlim,binw,xtk,xlbl,fname,filtw)

saveppt2('ppt',ppo);

close; %copy the figure created by rPSTH to the powerpoint.

end

saveppt2(pptName,'ppt',ppo,'save'); %save and close the powerpoint.

%Finish packaging Data for output.

Data(1).ytk=ytk; Data(1).StmLst=StmLst;

save([strrep(fname,'.mat','_data.mat')],'Data');
